# Pseudoprogression and hyperprogression during immune checkpoint inhibitor therapy for urothelial and kidney cancer

**DOI:** 10.1007/s00345-018-2264-0

**Published:** 2018-03-16

**Authors:** Francesco Soria, Andrea I. Beleni, David D’Andrea, Irene Resch, Kilian M. Gust, Paolo Gontero, Shahrokh F. Shariat

**Affiliations:** 1 Department of Urology and Comprehensive Cancer Center, Vienna General Hospital, Medical University of Vienna, Waehringer Guertel 18-20, 1090 Vienna, Austria; 2 Division of Urology, Department of Surgical Sciences, San Giovanni Battista Hospital, University of Studies of Torino, Turin, Italy; 3 Department of Urology, University of Texas Southwestern Medical Center, Dallas, USA; 4 Department of Urology, Weill Cornell Medical College, New York, USA; 5 Karl Landsteiner Institute of Urology and Andrology, Vienna, Austria

**Keywords:** Pseudoprogression, Hyperprogression, Immunotherapy, Treatment beyond progression, Checkpoint inhibitor

## Abstract

**Objectives:**

A small subset of patients treated with immune checkpoint inhibitors manifest atypical patterns of response, the so-called pseudoprogression (PP) and hyperprogression (HP). Their prevalence in urothelial (UC) and renal cancer (RCC) remains, to date, mostly uninvestigated. Therefore, we aimed to provide a summary of the current knowledge about PP and HP during immune checkpoint inhibitor therapy in UC and RCC patients.

**Methods and materials:**

A systematic medline/pubmed^©^ literature search was performed. The atypical patterns of response to systemic immunotherapy were reviewed. Endpoints were PP and HP in UC and RCC.

**Results:**

Tumors respond differently to immunotherapy compared to systemic chemotherapy. To evaluate response to immunotherapy, new guidelines (iRECIST) have been developed. To date, no studies focused on PP in UC and RCC, and the only way to evaluate its role is to take patients who respond to treatment beyond progression as surrogate for pseudoprogressors. PP seems to occur in a non-negligible rate of UC and RCC (from 1.5 to 17% and from 5 to 15%, respectively). The concept of HP, defined as a rapid progression after treatment, just took the first steps, and therefore, data from ongoing trials are awaited to elucidate its impact in genitourinary cancers.

**Conclusions:**

PP and HP are not uncommon entities in UC and RCC patients, treated with PD-1/PD-L1 inhibitors. Further investigation is warranted to define which patients are likely to experience PP and could benefit from treatment beyond progression and which ones will instead rapidly experience progression despite treatment and should, therefore, avoid systemic immunotherapy.

## Introduction

Over the last 5 years, immunotherapy has come to the forefront of cancer therapy, promising to change the treatment paradigms of advanced tumors. In the urologic context, the recent approval of multiple programmed death receptor-1 (PD-1) axis inhibitors is continuously transforming the treatment of advanced urothelial (UC) and renal cell carcinoma (RCC) [1], awakening hope where there was none.

At the same time, this represents a great challenge to physicians facing agents with novel mechanisms of action that differs from conventional chemotherapy and is unique in related side effects and patterns of response. One of the major challenges is actually the appropriate assessment of treatment response. It is known that tumors respond differently to immunotherapy compared to systemic chemotherapy and usage of traditional response evaluation criteria for solid tumor (RECIST) could result in tumor response misclassification [2].

A small subset of patients treated with immune checkpoint inhibitors manifest atypical patterns of response, the so-called pseudoprogression (PP) and hyperprogression (HP). The first, also known as tumor flare, is characterized by a transient increase followed by a decrease in total tumor burden [3]. HP instead is defined as a rapid increase in tumor growth rate (minimum twofold) compared to the expected growth rate [4]. These atypical patterns of response have been also reported for advanced UC and RCC, mainly as case reports in the context of phase II–III trials.

Understanding and identifying PP and HP is of fundamental importance for uro-oncologists to improve treatment decisions and patients’ outcomes. These phenomena are likely to be different from one malignancy to the others in addition to individual differences.

We sought, therefore, to review the literature to provide a summary of the current knowledge about PP and HP during immune checkpoint inhibitor therapy in UC and RCC patients.

## Methods and materials

A systematic medline/pubmed^©^ literature search was performed with different combinations of terms as “pseudoprogression”, “hyperprogression”, “response”, “radiological response”, “treatment beyond progression”, “bladder cancer”, “urothelial cancer” and “renal cancer”. Moreover, all the published RCTs enrolling patients with either UC or RCC were reviewed for the purpose of this article. No time period restriction was set. Original articles, reviews and editorials were selected based on their clinical relevance. Cited references from selected articles were analyzed to find and include significant papers missed from our search. The atypical patterns of response to systemic immunotherapy were reviewed. End points were PP and HP in UC and RCC.

## Summary of evidence

### How to evaluate the response to systemic immunotherapy

Immunotherapy does not exhibit the same patterns of response in comparison to traditional chemotherapy. Using assessment tools that have been developed and evaluated for conventional chemotherapy can result in inaccurate interpretation of the response, premature termination of therapy and unnecessary removal of patients from clinical trials, depriving them from a potentially life-extending treatment.

Historically, the World Health Organization (WHO) and the RECIST group have developed response criteria for solid tumors treated with systemic chemotherapy to standardize the characterization of treatment efficacy and to allow comparison between trials and with historical data [5, 6]. RECIST guidelines have been revised and a new version (RECIST 1.1) was published in 2009. Based on these recommendations, an early increase in tumor growth and/or the appearance of new lesions after therapy are considered as tumor progression and indicate the need for treatment cessation [7].

However, both WHO and RECIST 1.1 criteria have proven to be inadequate for the assessment of response to immunotherapy agents such as immune checkpoint inhibitors, partly because of the time needed to mount an antitumor immune response, and partly because of the possible occurrence of atypical patterns of response in subjects treated with immunotherapy.

In 2009, the immune-related response criteria (irRC) were proposed to specifically assess tumor response to immune checkpoint inhibitors with two main innovations: new or enlarged lesions should be incorporated into total tumor volume rather than immediately taken as indicator of disease progression; designation of disease progression requires an increase in tumor volume that has to be confirmed in two consecutive imaging studies at least 4 weeks apart [8].

In early 2017, RECIST working group developed a guideline of a modified RECIST 1.1 for immune-based therapeutics (iRECIST) [2]. Patterns of response based on iRECIST criteria include complete response (total remission of all target and non-target lesions, including the lack of appearance of new lesions; to be confirmed no less than 4 weeks after the first assessment); partial response (a decrease of at least 50% in the total tumor burden compared to baseline; to be confirmed after at least 4 weeks); stable disease (the change of the total tumor burden is reduced of less than 50% when compared with baseline or increased less than 20% compared to nadir); unconfirmed progressive disease (increase in the total tumor burden of at least 20% compared to nadir; further confirmation at imaging is needed to rule out PP); progressive disease (increase in the total tumor burden of at least 20% when compared to nadir confirmed by a further progression after 4–8 weeks) [9]. This update incorporates the suggestion of performing biopsies of target lesions whenever possible before the designation of relapse to rule out the presence of an immune infiltrate. In case of unfeasible biopsy, a follow-up scan should be performed to confirm relapse in all clinically stable patients. However, so far no clinical trials have incorporated biopsies of target lesions as part of their protocols.

Similar to RECIST 1.1 and irRC, also with iRECIST, treatment response evaluation could be done with almost all current imaging modalities including CT, MR and PET/CT. However, the measurements performed with iRECIST criteria seem to be more reproducible compared to the bidimensional approach used for irRC [9, 10].

### Atypical pattern of response and progression to systemic immunotherapy

With the widespread implementation of immunotherapy in the treatment paradigm of several malignancies, clinicians are facing a major challenge in the evaluation of treatment response. Recently, with the advent of immunotherapy, new response and progression patterns have been reported. Wolchock et al. [8] first provided a description of response patterns to systemic immunotherapy in patients with melanoma.

Pseudoprogression was first described in three patients with metastatic, unresectable melanoma treated with ipilimumab, a human anti-CTLA-4 monoclonal antibody [11]. These patients experienced an initial increased size of tumor lesions, with subsequent tumor burden and durable response. The histopathology of lesion biopsies confirmed the absence of tumor with the presence of inflammatory infiltrate or necrosis. Actually, it seems that PP is connected with infiltrations of active T cells and other immune cells within the lesion. According to immune-related response criteria (irRC), Hodi et al. [12] found that 12% of melanoma patients treated with pembrolizumab experienced PP before clinical and radiological responses. This atypical response was then confirmed to be present in several other solid tumors treated with PD-1 and PD-L1 inhibitors such as bladder, breast, colorectal, esophageal, gastric, head and neck, lung, pancreatoduodenal, ovarian, renal cell, sarcoma, and uterine cancer [13]. A systematic review of 38 studies found a 6% rate of atypical responses (151 of 2400 patients with solid tumors treated with anti-PD-1 therapy) [14].

Accurate identification of PP is of meaningful importance in decision making for treatment continuation. Moreover, its occurrence, together with a potential delayed effect of immunotherapeutic drugs, could account for the oncological results obtained in certain patients with the so-called “treatment beyond progression”, which is today allowed in clinical trials when specific criteria such as acceptable performance status, no impending end organ damage and no ongoing or serious toxic effects are met. Finally, PP seems to be associated with better overall survival (OS) rates (high likelihood of > 1 year survival) compared to patients experiencing progressive disease, stable response or partial response [15].

On the other hand, a new pattern of progression has been recently reported. It has been called HP and concerns a rapid disease progression under immunotherapeutic agents. Champiat et al., in a study of 218 patients with solid tumors or lymphoma treated either with PD-1/PD-L1 inhibitors, first reported the presence of hyperprogressive features in 12 (6%) immunotherapy-treated patients [4]. HP was defined as twofold increase of the expected tumor growth rate (which is an estimation of the increase in tumor volume over time) in patients with disease progression; it was associated with advanced age at treatment and worse oncological outcomes such as median overall survival (OS). Kato et al. [16] investigated the presence of potential genomic markers associated with HP in 155 patients with stage IV solid tumors treated in clinical trials with CTLA-4, PD-1/PD-L1 inhibitors or other investigation immunotherapeutic agents. Patients who suffered from HP harbored EGFR or MDM2/4 alterations, suggesting particular caution in treating patients with these genomic profiles.

However, the concept of HP is still unrefined suffering from limitations such as the low number of patients investigated, the use of unvalidated criteria to define patients who experience HP and the apparent absence of underlying mechanisms, difficult to understand in the absence of biopsy specimens from hyperprogressive lesions [17]. Therefore, data from ongoing trials are awaited to confirm this preliminary hypothesis, and in case, to solve this issue as quickly as possible.

#### Pseudoprogression in urothelial cancer

The frequency and clinical impact of PP in UC is almost uninvestigated. However, evaluating the number of patients who experience delayed response after treatment, the rate of PP seems to be lower if compared to other solid tumor patients. To date, no studies focused on PP and the only way to evaluate its role in urothelial cancers is to take patients which respond to treatment beyond progression as surrogate for pseudoprogressors (Table 1).

**Table 1:** List of studies investigating the treatment beyond progression as surrogate for pseudoprogression after immunotherapy in urothelial cancer

Refs.	Study design	Patients	Treatment	Number of patients	Evaluation criteria	Conventional response	Atypical response
Rosenberg et al. [16]	Phase II	Progressing metastatic urothelial cancer following platinum-based chemotherapy	Atezolizumab	310	RECIST 1.1	45	20
Sharma et al. [18]	Phase I/II	Progressing metastatic urothelial cancer following platinum-based chemotherapy	Nivolumab	78	RECIST 1.1	19	9
Sharma et al. [19]	Phase II	Progressing metastatic urothelial cancer following platinum-based chemotherapy	Nivolumab	265	RECIST 1.1	52	24
Powles et al. [17]	Phase I	Progressing metastatic urothelial cancer following systemic chemotherapy	MPDL3280A	67	RECIST 1.1	17	1

In the IMvigor210 phase II trial, locally advanced and metastatic urothelial cancer patients who have progressed following treatment with platinum-based chemotherapy were treated with atezolizumab [18]. Overall, 121 of 310 patients were treated beyond progression and 20 (6%) of them experienced a delayed response (defined as subsequent ≥ 30% decrease in volume lesion from baseline) despite initial radiological progression. Previously, Powles et al. [19] reported a PP rate of 1.5% in patients treated with atezolizumab.

Atypical patterns of response have been seen also in patients resistant to platinum-based chemotherapy who received nivolumab. In the phase I–II CheckMate 032 trial, nine patients (12%) experienced delayed response after initial progression [20]. Similarly, of 265 patients who received nivolumab in the CheckMate 275 study [21], 70 (26%) were treated beyond progression and 24 (9%) experienced non-conventional benefits. Non-conventional benefiter patients had to meet one of the following criteria: appearance of a new lesion followed by decrease from baseline of at least 10% in the sum of the target lesions; initial increase of the target lesions followed by reduction from baseline of at least 30%; initial increase of the target lesions followed by at least two tumor assessments showing no further progression. Unfortunately, the survival data of patients experiencing non-conventional patterns of response in these trials are, to date, not available.

Finally, no data about PP in patients with metastatic urothelial cancer treated either with pembrolizumab, avelumab or durvalumab are reported so far.

#### Pseudoprogression in renal cancer

Although RCC has been treated with immunomodulating agents such as cytokines for nearly 40 years, the imaging patterns and features of response to the new anti-PD-1/PD-L1 agents need further characterization. No study specifically focusing on atypical patterns of response in renal cancer have been published so far. Moreover, biopsies of target lesions were not performed in any of the trials investigating the effect of immunotherapeutic agents in metastatic RCC (mRCC). Therefore, the occurrence of PP in RCC could only be estimated by evaluating the proportion of patients treated beyond progression who experienced a sustained reduction and/or stabilization in the size of target lesions despite initial progression (Table 2).

**Table 2:** List of studies investigating the treatment beyond progression as surrogate for pseudoprogression after immunotherapy in renal cell carcinoma

Refs.	Study design	Patients	Treatment	Number of patients with mRCC	Evaluation criteria	Conventional response	Atypical response
Brahmer et al. [20]	Phase I	Metastatic solid tumors refractory to systemic treatment	Nivolumab	1	RECIST 1.0	0	1
McDermott et al. [22]	Phase I	Metastatic renal cell refractory to systemic treatment	Nivolumab	34	RECIST 1.0	9	3
Motzer et al. [23]	Phase II	Progressing metastatic renal cell carcinoma following VEGF inhibitors	Nivolumab	168	RECIST 1.1	35	25
Escudier et al. [24]	Phase III	Metastatic renal cell refractory to systemic treatment	Nivolumab	406	RECIST 1.1	90	20

Within a phase I study, Brahmer et al. first reported the case of a 72-year-old with multiorgan mRCC who experienced an atypical response after one cycle of nivolumab; progression in a pancreatic metastasis but regression in other sites. This evolved to an overall partial response after two additional cycles, lasting over 16 months without further therapy [22]. In an analog phase I, dose-escalation study of 296 patients with solid tumors, a total of eight patients (3%) with melanoma, lung cancer, or RCC, experienced PP [23]. In the related cohort expansion study of 34 mRCC patients, up to 29% (*n* = 9) of objective responses, as per RECIST criteria, were observed. Three additional patients (9%) experienced unconventional responses that did not fit RECIST criteria such as persistent reduction in target lesions in the presence of new lesions or regression after initial progression [24].

Recently, in a randomized phase II trial investigating the effect of different nivolumab doses (0.3, 2 or 10 mg/kg) in mRCC patients previously treated with agents targeting the vascular endothelial growth factor pathway, 36 patients were treated beyond progression and 25 of them (69%) experienced durable response consisting in tumor reduction or stabilization [25].

These findings led to the idea that patients treated with nivolumab could potentially benefit from treatment beyond progression. Based on this hypothesis, Escudier et al. [26] investigated the clinical benefit of treatment beyond progression in the context of the phase III CheckMate 025 study (NCT01668784). Of the entire cohort of 406 enrolled patients, 316 (78%) progressed and 153 of these (48%) were treated beyond progression. The choice of continuing treatment despite progression was based on patients’ and tumor’s characteristics such as good performance status, short time to progression, low incidence of new bone lesions and improved quality of life. An objective response, defined as ≥ 30% of tumor burden reduction, was observed in 20 (13%) patients. To date, there are no available data about survival in patients treated beyond progression who experienced a subsequent response.

#### Hyperprogression in urothelial and renal cancer

Hyperprogression is a very poorly investigated phenomenon. To our knowledge, only two studies focusing on this atypical pattern of response have been published so far, and only one trial enrolled patients with UC and RCC [4, 27]. Champiat et al. [4], by reviewing the medical records of 218 patients with solid tumors treated with immunotherapy, aimed to investigate prevalence, natural history and predictive factors of HP. Of the 131 evaluable patients, 12 (9%) were considered as having HP, defined as ≥ twofold increase of the tumor growth rate after anti-PD-1/PD-L1 therapy. Two out of eight (25%) patients with urothelial cancer enrolled in the study experienced HP, representing one of the highest proportions among all cancer types. On the other hand, no patients with RCC were found to rapidly progress after treatment. Older age at treatment was found to be associated with a higher risk of developing HP.

Despite the low number of patients evaluated and the mainly informative nature of the study, this trial points out that although immunotherapy represents a breakthrough by leading to durable tumor response in some cases, a small subset of patients seem to experience a tumor flare after treatment, leading inevitably to death.

## Conclusions

Till now, the occurrence of PP in metastatic UC and RCC has been only suggested by evaluating patients treated beyond progression who finally experienced sustained reduction in tumor burden or stabilization in the size of target lesions. However, the efficacy of treatment beyond progression could be explained by several factors. First, the occurrence of the PP phenomenon; second, the presence of late responder patients (who require a longer period of time on treatment); third, the presence of the genetic intratumor heterogeneity that could be involved in the case of mixed response (reduction of target lesion in the presence of new metastatic deposits).

To our knowledge, biopsies of target lesions in patients with metastatic UC and RCC to histologically demonstrate the presence of an immune infiltrate have never been performed. Therefore, to better understand the real incidence of PP in these patients, future trials should include biopsies of target lesions as part of the protocol, as suggested by iRECIST guidelines. Moreover, further investigations are warranted to better define which patient will respond to treatment beyond progression to provide better survival outcomes without compromising safety and quality of life, which remains one of the main target to pursuit in metastatic and end-of-life settings.

Hyperprogression remains an almost undiscovered entity. Further studies are urgently needed to understand why some patients rapidly progress after treatment and predicting which patient will experience this undesired outcome.
